# Impact of an educational intervention on hand hygiene practice among nursing students, with a focus on hand drying efficacy

**DOI:** 10.1177/17571774231224695

**Published:** 2023-12-28

**Authors:** John Gammon, Julian Hunt, Lisa Duffy, Ioan Humphreys, Jon Hinkin, Alan Watkins

**Affiliations:** 1 School of Health and Social Care, 7759 Swansea University, Swansea, United Kingdom of Great Britain and Northern Ireland; 2 Department of Nursing, 7759 Swansea University, Swansea, United Kingdom of Great Britain and Northern Ireland; 3 Health and Wellbeing Academy, 7759 Swansea University, Swansea, United Kingdom of Great Britain and Northern Ireland; 4 Biomedical Sciences, 7759 Swansea University, Swansea, United Kingdom of Great Britain and Northern Ireland

**Keywords:** Nursing students, educational intervention, hand hygiene, hand drying, nursing education

## Abstract

**Background:**

Hand hygiene and its significance for reducing the spread of infection is well evidenced and has been brought into sharp focus following the COVID-19 pandemic. Although a crucial clinical skill in ensuring safe healthcare, little is known regarding nursing students’ effectiveness of hand hygiene practice.

**Aim:**

The aim of this study was to evaluate the impact of an educational intervention on hand hygiene practice, designed by the research team for first year pre-registration nursing students. Particular emphasis was placed upon hand drying technique and time.

**Methodology:**

825 nursing students were observed and assessed for their hand hygiene practice in a clinical suite at a university setting. Nursing students were observed for compliance against set outcome measures involving hand hygiene preparation, hand and wrist washing technique, hand drying technique and time. Data were analysed quantitatively using SPSS.

**Results:**

The educational intervention had a significant impact on the clinical skills learning of nursing students. 779 students passed the assessment at the first attempt (94.4%). Of the 46 students that failed to meet the necessary criteria, 45 satisfied the criteria at the second attempt; giving an overall optimal compliance of 99.9%. 99.6% of students complied with recommended hand drying standards.

**Conclusion:**

This study offers an important contribution to the development and delivery of nursing education programmes. The educational intervention improved compliance with recommended hand hygiene technique and practice. Lack of attention to hand drying may negate effective hand hygiene in healthcare.

## Introduction

Hand hygiene and its efficacy in controlling the spread of infection is well evidenced. Research shows that hand hygiene is one of the more significant strategies for preventing the spread of microorganisms (Luangasanatip et al., 2015; Schweizer et al., 2014) and is applicable to practitioners and the public in controlling the spread of disease. The role of hand hygiene as an important strategy in controlling the spread of infection has been brought to light following the Coronavirus (COVID-19) pandemic (Alzyood et al., 2020; Loftinejad et al., 2020). Hand hygiene has been a pivotal behavioural control strategy forming a vital part of the international response to the global emergency (Centers for Disease Control and Prevention, 2020, World Health Organization [WHO], 2020).

Promotion of improved hand hygiene is recognised as being a crucial measure in public health and is an essential skill of pre-registration nurse education and professional development for registered nurses. However, whilst hand hygiene is cost effective and highly efficient in controlling the spread of microorganisms, research suggests that the level of knowledge, skills, experience and compliance of nursing and medical students, and healthcare professionals, is suboptimal, and does not reflect expected standards of practice (Awaji and Al-Surimi, 2016; Azim et al., 2016; Jeong and Kim 2016; Kingston et al., 2018; Labrague et al., 2018; Oncu et al., 2018; Walaszek et al., 2017). Moreover, the emphasis of public health campaigns and practitioner education has been on hand washing and hand sanitising with alcohol-based hand sanitisers, with little emphasis on hand drying practice.

Hand drying is integral to hand hygiene, and consequently the correct drying of hands after washing should be an essential component of hand hygiene education and practice for all healthcare practitioners (Gammon and Hunt, 2018). Nonetheless, global campaigns, including World Hand Hygiene Day campaigns such as the World Health Organization’s (WHO) SAVE LIVES—Clean Your Hands and the Centers for Disease Control and Prevention (CDC) Clean Hands Count Campaign, as well as much of the existing nursing research, focus on the efficacy of nurses’ hand washing behaviour (Gammon and Hunt, 2018) and thus the extent of our understanding of which educational interventions for students are effective in influencing and changing professional behaviour around hand hygiene, remain limited. Therefore, the development of an educational intervention that provides nursing students with the knowledge and understanding of each stage of the hand hygiene process with consistent assessment is viewed as offering an important contribution in developing and delivering effective infection prevention and hand hygiene in nursing education programmes.

## Background

Learning a clinical skill is a complex, yet essential, element of nursing programmes; in order to ensure nursing students become confident, safe and effective practitioners. Effective learning of clinical nursing skills involves engaging nursing students in teaching strategies that are practical, affective and cognitive (Christiansen and Bell, 2010; Ghasemi et al., 2020). Haggman-Laitilia et al. (2016) suggest that educational interventions should provide nursing students with sufficient competencies for implementing all learning categories of evidence-based nursing, with specific focus on the implementation of evidence in patient care and on medium to long term effectiveness. The influence of different teaching and learning strategies, as well as learning contexts and settings, such as academic and clinical settings, are particularly important (Ghasemi et al., 2020; Haggman-Laitilia et al., 2016). In Stacey et al.’s (2018) study, the integration of the Knowledge and Understanding Framework within the wider training programme provided pre-registration mental health nursing students with an opportunity to understand themselves as clinicians; shifting their perceptions away from focussing on the perceived ‘difficult’ behaviour of a client towards an understanding of their own emotional responses to the behaviours. The framework utilised in this research was similar to that applied to the context of hand hygiene in this study.

The aim of the present study was to evaluate the ways in which an educational intervention tool, designed by the research team, impacted on pre-registration nursing students’ hand hygiene practice; which included emphasis on hand drying and the ways in which educational interventions can support the efficacy of this aspect of hand hygiene.

## Research methods

The research design of this study was evaluative, utilising non-participatory observation, in order to understand and assess the impact of the educational intervention and assessment tool on hand hygiene amongst nursing students.

Data was collected as part of nursing students’ assessment; therefore, ethical approval was not required.

### Educational intervention

The hand hygiene educational intervention (see: Box 1) was developed as a result of a scoping review of existing literature, a synthesis of current national and international guidelines, the clinical experience of the researchers and the academic expertise within the research site. This resulted in a localised educational intervention which aimed to address the identified research gaps and issues of practice compliance with regards to hand hygiene. In particular, identified gaps included the lack of valid measurement tools to assess hand hygiene skills and technique and limited evidence on measuring hand drying efficacy. No theoretical framework was used to inform the educational intervention.

### Assessment tool

The study used an Objective Structured Clinical Examination (OSCE) as the means of data collection and assessment. OSCE is an established educational method of facilitating learning and assessment of students clinical skills in nursing education (Meskell et al., 2015). The educational intervention was utilised to underpin and inform the summative assessment (see: Box 2).

The study considered that skills assessment tools are criterion referenced tools and need to measure against specific criteria or standards (Cant et al., 2013). The criteria for the hand hygiene assessment were produced by using a process of ‘blue-printing’ from evidence-based standards and expert opinion (Loveday et al., 2014). The hand hygiene technique was ‘mapped’ from the WHO (2009) steps. However, in WHO’s sequence, wrists are omitted. The assessment tool utilised in this study was adapted to include wrists to the process. The evaluation of the observed technique and skills performance of nursing students’ was undertaken against the agreed hand hygiene assessment outcomes illustrated in Box 2.

## Box 1: Hand hygiene educational intervention

**Table:** 

Phase	Mode of delivery		Learning outcome
1: Within first month of infection prevention and control (IPC) module	Three consecutive infection prevention and control (IPC) lead lectures		Introduction to IPC, standard IPC precautions and importance of effective hand hygiene
	Introduction to infection (IPC) (introduces the importance of hand hygiene)	(1 hour) 12 – 200 students	
	Introduction to IPC 1—food hygiene, environmental cleanliness	(1 hour)	
	Introduction to IPC 2—standard precautions includes handwashing theory and practice	(2 hour)	
2: Within first month of the IPC module following lead lectures	Practical interactive teaching session, in small groups of students <25. (1 h)		Observe and practice skill of effective handwashing. Use of ‘glo and tell’ visual assessment tool
3: Within second month of the IPC module	Formative assessment		Students observed, assessed and immediate feedback given on ability to perform correct handwashing technique
4: At end of second month prior to first clinical placement	Summative practical assessment: Hand hygiene intervention		Students assessed pass (optimal) or fail (suboptimal) on the performance of all elements of this practical component

## Box 2: Hand hygiene assessment tool

**Table:** 

	Component of the skill	Pass	Fail
A	The student is ‘Bare below the Elbows’. Watches, bracelets and rings, except a plain band ring, are removed. No nail varnish, nail jewels and/or false nails worn. Nails should be short and clean. Sleeves are short or rolled up. All skin abrasions/cuts should be covered with a waterproof dressing		
B	Set a comfortable water temperature before commencing hand washing (tepid)		
1	Wet hands under running water before applying soap		
2	Apply sufficient soap to cover all hand surfaces		
3	Rub hands palm to palm vigorously		
4	Rub back of each hand with palm of other hand with fingers interlaced		
5	Rub palm to palm with fingers interlaced		
6	Rub with back of fingers to opposing palms with fingers interlocked		
7	Rub each thumb clasped in opposite hand using a rotational movement		
8	Rub tips of fingers in opposite palm in a circular motion		
9	Wash wrists		
C	Steps 3–9 should take at least 15 s		
10	Rinse off soap from all surfaces thoroughly		
11	Turn off tap without contaminating hands		
12	Dry all surfaces of hands thoroughly with a single-use disposal paper towel		
D	Dispose of paper towels correctly, using pedal of pedal bin		
E	Student completes sequence without re-contaminating hands up to point C (unless student recognises error and recommences skill)

### Research setting and participants

This study involved a total number of 825 pre-registration nursing students studying at a university in Wales, UK. All were first year students and were at Progression Point 1 of the undergraduate nursing programme. As part of the educational intervention, all nursing students had received a lead lecture relating to infection prevention, hand hygiene and attended a practice skills teaching session on hand hygiene technique. Each student attended a formative hand hygiene assessment session where they were observed and provided with immediate verbal feedback in relation to their performance. A non-probability, purposive sample was used, allowing data to be collected that was representative of the target population and provided the volume of data required for analysis.

### Data collection

Nursing students were observed for their hand hygiene practice in a clinical practice suite at a university setting. This involved the washing of hands and wrists with soap and water, and the drying of hands with a single-use disposable paper towel. The hand hygiene assessment tool provided a means of reducing observer bias and maintaining a level of objectivity in the evaluation of technique and skills performance. Data collection followed epic3 guidelines (Loveday et al., 2014). Nursing students were observed, assessed and video recorded individually as a means of ensuring reliability and validity. Electronic software facilitated the recording, storage and analysis of each OSCE. The use of the electronic OSCE Management Information System (MIS) offered time savings, enabled benchmarking and assisted with further moderation processes of reliability and consistency (Meskell et al., 2015).

In maintaining objectivity, nominal data were collected using a simple electronic rating of performance by scoring or Yes/No for each criteria of the hand hygiene assessment tool. Assessment forms were completed by study researchers, submitted electronically via MIS and later obtained by the full research team.

As part of the research, and in line with the University’s moderation policy, all suboptimal assessments were further moderated by an external examiner; together with a random selection of optimal assessments.

### Data analysis

Statistical analysis was undertaken in SPSS Version 22 for Windows. The relation between assessment outcome, cohort and year was analysed using binary logistic regression. We used one-sample Kolmogrov–Smirnov tests to assess whether data on ‘optimal’ and ‘suboptimal’ compliance satisfied specific distributional (uniform, Normal) assumptions; where it did not, the Wilcoxon Signed Rank Test, an appropriate non-parametric equivalent test, was applied. All tests were summarised by *p*-values, with *p* < .05 regarded as statistically significant.

## Results

Following the educational intervention, the Hand Hygiene Assessment results (Table 1) were grouped by point of intake (March, September and September Part Time), the outcome of the assessment (Optimal Practice or Suboptimal Practice) and by year (Table 2). The number of suboptimal compliance scores indicate an individual nursing student and not the number of stages of the intervention their practice scored as suboptimal. Logistic regression analysis is shown in Table 3, with the breakdown of each Stage suboptimal assessment score is shown in Table 4.

**Table 1.** Hand hygiene assessment—results grouped by cohort and year.

	Year				
Point of Intake	Outcome	2016	2017	2018	Total
March	Optimal	67	80	100	247
	Suboptimal	13	8	5	26
	Total	80	88	105	273
September	Optimal	258	255	—	513
	Suboptimal	7	13	—	20
	Total	265	268	—	533
September	Optimal	—	19	—	19
Part time	Suboptimal	—	19	—	19
Total	Optimal	325	354	100	779
	Suboptimal	20	21	5	46
	Total	345	375	105	825

**Table 2.** Outcome of hand hygiene assessment—summary table by cohort.

Cohort	Number of Pre-Registration Students	Number of Optimal	Number of Suboptimal: 1st Attempt	Percentage of 1st Attempt Suboptimal (%)	Number of Suboptimal: 2nd Attempt	Number of Optimal: 2nd Attempt	Percentage of 2nd Attempt Suboptimal (%)	Percentage at 1st Attempt (%)	Percentage at 2nd Attempt (%)
M16	80	67	13	16.25	0	13	0.00	83.8	100.0
S16	265	258	7	2.64	0	7	0.00	97.4	100.0
M17	88	80	8	9.09	0	8	0.00	90.9	100.0
S17	268	255	13	4.85	1	12	7.69	95.1	99.9
M18	105	100	5	4.76	0	5	0.00	95.2	100.0
S17PT	19	19	0	0.00	0	0	0.00	100.0	100.0
Total	825	779	46	5.58	1	45	2.17	94.4	99.9

Abbreviations: M – March and S – September. 16, 17 and 18 – Year of cohort intake.

**Table 3.** Logistic regression analysis of the hand hygiene assessment results.

		B	S.E.	Wald	Df	Sig	Exp (B)	95% C.I.for EXP(B)	
								Lower	Upper
Step 1^a^	Year	−0.378	0.223	2.892	1	0.089	0.685	0.443	1.059
	Cohort	−1.224	0.324	14.257	1	0.000	0.294	0.156	0.555
	Constant	761.048	448.797	2.876	1	0.090			

^a^Variable(s) entered on step 1: Year/Cohort.

**Table 4.** Suboptimal points of hand hygiene assessment.

	Suboptimal points of assessment																
Cohort	A	B	1	2	3	4	5	6	7	8	9	C	10	11	12	D	E
M16	0	0	0	0	3	0	0	1	0	8	0	0	2	1	1	0	0
S16	0	0	0	0	0	0	1	0	1	3	0	0	1	1	0	0	0
M17	0	0	1	1	0	1	2	1	1	1	1	0	0	0	2	1	0
S17	1	0	0	0	0	0	0	0	2	5	1	0	2	0	0	1	1
M18	0	0	1	0	0	0	0	0	1	2	0	0	1	0	0	0	0
S17PT	0	0	0	0	0	0	0	0	0	0	0	0	0	0	0	0	0
Total	1	0	2	1	3	1	3	2	5	19	2	0	6	2	3	2	1

Only the first attempt at the assessment was analysed, as the low numbers of suboptimal compliance scores at the second attempt would have rendered analysis statistically meaningless. Due to the low numbers in the September Part Time cohort, it was decided to remove their results from the analysis and not make the distinction between full and part time. In addition, a non-parametric test (Wilcoxon Signed Rank Test) was undertaken on the data to compare the ‘optimal’ and ‘suboptimal’ compliance scores. The resulting differences were seen to be statistically significant (*p*-value = <0.001).

Table 2 shows a summary of the cohort intakes and the percentage optimal rate at each attempt of the assessment. Each nursing student was permitted two attempts at completing the assessment, with a fourteen-day interval between attempts. There was an overall optimal compliance rate of 94.4% across the six cohort intakes. One nursing student scored suboptimal at the second attempt of the assessment (S17), giving an optimal compliance score of 99.9% across the six cohort intakes.

Logistic regression was conducted by year and by cohort (March and September) for the first attempt. The results of the logistic regression analysis (Table 3) shows that there were fewer suboptimal scores seen in point of intake September compared to point of intake March and the difference was seen as statistically significant (*p*-value <.001). Further regression analysis showed that the variable ‘Year’ had no impact on the difference. That is, there was no year on trend seen (Table 3).

Table 4 shows the 15 stages of the OSCE and the number of suboptimal scores at each stage. It was observed that a large number of the suboptimal practice appears at Stage 8 (36%), Stage 7 (9%) and Stage 10 (11%). Cohort M16 saw the highest suboptimal scores at Stage 8 with 50% of the students failing at the first attempt. Cohort S16 shows a failure rate of 43% and cohort M18 a rate of 40%.

Initial observation of the suboptimal stages of the assessment saw a marked suboptimal rate at Stage 7 and at Stage 8. In light of this, a one-sample Kolmogrov–Smirnov Test for uniformity was conducted on the assessment results. This test showed a statistically significant (*p*–value = <0.002) result for non-uniformity of scores at each stage of the hand hygiene intervention (Table 5). Therefore, non-uniformity can be assumed as a result of the 36% suboptimal compliance score at Stage 8.

**Table 5.** One-sample Kolmogrov–Smirnov test to test for uniformity.

One-sample Kolmogorov–Smirnov Test		Fail Section
N		53
Uniform Parametersa,b	Minimum	1
	Maximum	17
Most Extreme differences	Absolute	0.255
	Positive	0.162
	Negative	−0.255
Kolmogorov–Smirnov Z		1.854
Asymp. Sig. (2-tailed)		0.002

^a^Test distribution is Uniform.

^b^Calculated from data.

## Discussion

The aim of this study was to evaluate the impact of a self-designed educational intervention on the efficacy of hand hygiene practice among first year pre-registration nursing students. The study considered the whole of the hand hygiene procedure with particular emphasis being given to hand drying practice. To our knowledge, there are no comparable studies within the literature.

Not all nursing students began their studies from the same position. The extent to which they had received hand hygiene mentorship as part of their previous roles prior to commencing their studies, is largely unclear. The exception of this are cohort S17PT, who were made up of healthcare support workers employed within the local health board and who scored 100% optimal hand hygiene practice. In this way, the variability in previous experience of working in clinical settings where nursing staff practice hand hygiene while developing nursing skills appears to be significant in effective hand hygiene practice.

In this study, nursing students overall optimal assessment score over the 3 years studied was 99.9%, with positive impact being most acute at the phase of preparation involving Stages A to 4 and at the drying phase, Stages 11 to E of the hand hygiene intervention. Of the overall optimal score, the optimal score at the first attempt of the summative assessment was 94.4% and 99.9% at second attempt. The statistical results suggest convincing and meaningful level of suboptimal practice within the phase of washing, Stages 5 to 10 and especially at Stage 8. These stages are viewed as being the most complex stages of the Hand Hygiene Assessment Tool, in that nursing students are required to demonstrate the correct positioning and movements of their hands. At Stage 8 in particular, the handwash solution needs come into contact with all of surfaces of the hands and the palms of the hands rubbed rotationally with the fingertips of the opposite hand. The rationale being that the fingertips need to be cleaned effectively in order to reduce the carriage of potential pathogens under the fingernails and render the hands of nursing students safe in clinical placement. Moreover, this research further found that there was no year on year statistical difference in scores between March 2016 and September 2018. There were, however, fewer suboptimal assessment scores at the September points of intake compared to March intakes. This difference was seen as being statistically significant.

Working memory is highly complex in that it can only process a limited number of information elements at any given time (Nicholls et al., 2016). This constraint creates a ‘bottleneck’ for learning (Young et al., 2014). Successful learning requires the interplay of multiple processes; including cognitive, affective, social, environmental and metacognitive domains (Young et al., 2014). In this way, the design principles of the four stage Educational Intervention (Box 1) utilises a phased approach involving opportunities for enhancing the capacity of working memory of nursing students through three infection prevention lead lectures, a practical interactive effective handwashing teaching session and a rehearsal formative assessment, prior to summative assessment. Following the formative assessment, nursing students are provided with immediate feedback; decreasing cognitive overload and thus improving the knowledge and understanding necessary in order for nursing students to perform the psychomotor skills unique to effective hand hygiene, and deliver competent and safe practice.

Hand drying is a much neglected aspect of hand hygiene, with little emphasis within nursing curricula or hospital policies of the efficacy of hand drying and hand drying practice; or indeed in clinical practice more widely (Gammon and Hunt, 2018). In these circumstances, this study, in considering every stage of the hand hygiene process, positions particular focus on the ways in which educational interventions can support the efficacy and assessment of hand drying practice among nursing students. This study demonstrates the positive impact of the hand hygiene educational intervention around hand drying, showing a high level of optimal scores (3/825%–99.6%) among nursing students at Stage 12 (hand drying stage) of the assessment. This is contrary to much of the existing literature which affirms hand hygiene amongst practitioners and nursing students to be suboptimal (Awaji and Al-Surimi, 2016; Azim et al., 2016; Jeong and Kim 2016; Kingston et al., 2018; Labrague et al., 2018; Oncu et al., 2018; Walaszek et al., 2017).

## Study limitations

Observation is considered to be the golden standard in the assessment of hand hygiene. In this study, nursing students were being observed and assessed within a controlled environment. Nursing students were aware that they were being observed and assessed, and this may have influenced their performance at the time. While our study presents important information regarding the moment specific practices of nursing students within a university clinical practice suite, that is nursing students’ practices at the point of assessment, this study does not reflect or replace nursing students’ hand hygiene practices while on clinical placements. In this way, the longer term effects of the educational intervention were not assessed in this study. Research suggests that one-off hand hygiene educational interventions have a short-term influence and without repeated reinforcement, the effect is not maintained over longer periods (Moore et al., 2021; Naikoba and Hayward, 2001; Price et al., 2018). Moreover, the sample of nursing students was collected from one higher education institution in the UK between March 2016 and September 2018, and therefore our results cannot necessarily be generalised to other healthcare students. Whether these students differ from the rest of the nursing student population in the UK is unknown. Objectivity in this study is maximised through the use of the hand hygiene assessment tool that included the agreed outcome measures. Nonetheless, subjective bias by the observer cannot be ruled out. In light of this, additional measures were put in place involving the application of moderation processes and review of the OSCEs by the external examiner.

## Implications for nursing education and hand hygiene practice

This study offers an important contribution to the broader understanding of hand hygiene practice among nursing students. Positioned within the context of the global Covid-19 pandemic, this study highlights the importance of effective hand hygiene nursing education and the efficacy of the utilised educational intervention in teaching nursing students the skill and technique of hand hygiene. The findings from this study may be used to further develop teaching methods to improve nursing students hand hygiene practice, support curriculum reform and offers insight in determining an optimal pedagogic approach to hand hygiene nursing education.

The method of summative assessment developed and utilised in this study meets the proficiency standards for registered nurses laid down by the Nursing and Midwifery Council (2018a, Nursing and Midwifery Council [NMC], 2010; Nursing and Midwifery Council [NMC], 2018b), and may inform educational strategies to maintain hand hygiene compliance during clinical placement and practice at hospital settings.

## Conclusion

The significance of hand hygiene for preventing the transmission of microorganisms and reducing the spread of infection has been brought into sharp focus following the COVID-19 pandemic. In these circumstances, nursing education must ensure that nursing students adopt optimal hand hygiene practice; being aware of when, how and why hand hygiene is essential and understanding the implications if adherences to hand hygiene are not followed. An effective learning process develops the construction of skills automation, involving free working memory to steer positive behaviour essential to safe and effective hand hygiene practice.

This study positions particular emphasis on hand drying. The significance of the role of hand drying has not been widely promoted, and its relevance to hand hygiene and infection prevention appears to have been neglected. Lack of attention to this aspect may negate the benefits of effective hand hygiene in healthcare. Although moment-specific, this study forms an initial step and first evaluation in understanding the types of nursing educational interventions that can support (or otherwise) optimal hand hygiene practice in nursing students and in examining the contribution that efficient hand drying makes to the overall effectiveness of hand hygiene practices among pre-registration nursing students. Further research could follow the continuing hand hygiene assessment of nursing students over the duration of their studies as they move toward clinical practice, thus observing as to whether compliance and effectiveness is sustained in the longer term.

To continually improve and maintain the optimum efficacy and compliance of nursing students hand hygiene practice, hand hygiene education should be emphasised during clinical placements, which entails increased collaboration between the university, hospital sites and nursing staff mentors. Increased collaboration may involve implementing hand hygiene interventions, goal-setting, regular monitoring and reinforcement of clinical practice skills, reward incentives, and timely and effective feedback and nudge behaviour prompts to ensure sustainability and greater improvements in hand hygiene and drying compliance. Nursing education is critical in shaping professional competence and nursing students make an important contribution to the delivery of healthcare. This study offers an important contribution in developing and delivering nursing education programmes and infection prevention in nursing curricula.
